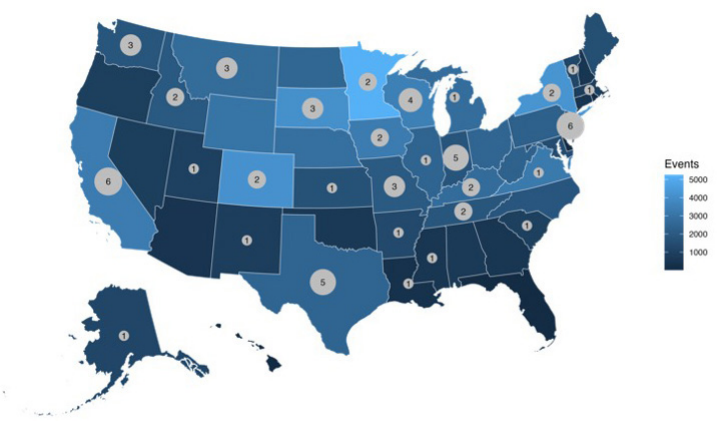# 955 Impact of Climate Change on Pediatric Frostbite and Cold-Weather Injuries

**DOI:** 10.1093/jbcr/iraf019.486

**Published:** 2025-04-01

**Authors:** Emily Colonna, Charly Vang, Rediat Tilahun, Lexy Kindt, Kyle Schmitz, Derek Lumbard, Rachel Nygaard

**Affiliations:** Hennepin Healthcare; Hennepin Health Care Research Institute; Hennepin Health Care Research Institute; Hennepin County Medical Center; Hennepin Healthcare; Hennepin Healthcare; Hennepin Healthcare

## Abstract

**Introduction:**

Extreme weather events are increasing in regions unaccustomed to freezing temperatures, placing more children at risk for frostbite and cold-related injuries, which can lead to significant morbidity and mortality. Many medical centers lack established frostbite management protocols, where early identification and treatment are essential. This study reviews pediatric cold-weather injuries across multiple databases to delineate the extent and management of these injuries and identify geographic areas where targeted intervention programs could be most impactful. We hypothesize that the utilization of multiple databases will allow us to identify specific geographic areas where targeted cold-weather intervention programs could be most impactful.

**Methods:**

A retrospective study of data from the National Oceanic and Atmospheric Administration Storm Event Database (NOAA) (2016-23), the National Readmission Database (NRD) (2016-20), and a hospital burn registry (2017-22). Pediatric patients (age < 18) diagnosed with frostbite or cold-related injuries were included. We analyzed the frequency of events, demographics, treatment, outcomes, and readmission rates.

**Results:**

We identified 11 pediatric frostbite admissions at our institution between the ages of 3 to 17 (64% >11 years). Treatment modalities included rapid rewarming, thrombolytics, and perfusion imaging, with no resulting amputations. We used the NRD to evaluate pediatric injuries nationally since standard management for severe frostbite injury includes an approximately one month delayed-planned readmission to promote preservation of digit length in cases where amputation is required. We identified 73 pediatric frostbite admissions with 15 classified as severe frostbite and 8 were treated with thrombolytic therapy. There were 10 readmissions, 4 amputations, and 5 were treated for cellulitis. Not all cold-related injuries, especially fatalities, result in hospital treatment, so we used the NOAA database to identify cold-weather event associated fatalities. We found 65 pediatric fatalities out of a total of 1122 total cold-related fatal events (Fig 1). Pediatric mortality did not coincide with highest or lowest cold-weather events.

**Conclusions:**

This is the first study to integrate multiple national databases to track pediatric cold-weather injuries and fatalities. This comprehensive understanding of regional risks identifies states where potential educational interventions could prevent pediatric cold-weather injury and fatality. Limitations to current public health data tracking underscores the necessity of integrating multiple data sources to comprehensively understand the impact of cold-weather injuries in the U.S.

**Applicability of Research to Practice:**

Tailored intervention programs coupled with cold-weather management protocols implemented at medical centers across the United States can safeguard children against the adverse effects of cold weather.

**Funding for the Study:**

N/A